# The influence mechanism of interlocking director network on corporate risk-taking from the perspective of network embeddedness: Evidence from China

**DOI:** 10.3389/fpsyg.2023.1062073

**Published:** 2023-03-02

**Authors:** Hua Li, Yangyang Li, Qiubai Sun

**Affiliations:** School of Business Administration, University of Science and Technology Liaoning, Anshan, China

**Keywords:** interlocking director network, corporate risk-taking, financing constraints, R&D investment, industry competition intensity

## Abstract

The interlocking director network can not only help achieve low-cost information sharing and exchange learning among enterprises, but also provide essential resource support for corporate risk-taking behavior. This study aims to empirically analyze the impact, mechanism of action, and boundary of influence of interlocking director network (NET) on corporate risk-taking (RISK) using data of Chinese A-share listed companies from 2007 to 2020.The results show: (1) There is a significant positive correlation between NET and RISK, and the above results are still established after a series of robustness tests. (2) Mechanistic tests show that the NET can promote RISK through two channels: alleviating financing constraints and increasing R&D investment. (3) Further analysis reveals the promotion of NET on RISK is more significant in non-state-owned enterprises and enterprises with higher industry competition intensity. These findings have positive implications for the construction of an inter-enterprise interlocking director network and the enhancement the of the risk-taking level.

## 1. Introduction

The outbreak of COVID-19 and the increasingly fierce global competition have brought unprecedented risks and challenges to the development of Chinese enterprises. In such an economic situation, enterprises must appropriately improve their level of risk-taking if they want to maintain competitive advantage and achieve long-term sustainable economic growth (Tran, 2019). Corporate risk-taking indicates the preference of enterprises for high-risk and high-return projects in the investment process, reflecting the analysis and selection of investment projects that can generate expected returns and cash flows but are fraught with uncertainty factors by enterprise managers (Min et al., 2015). The higher the level of corporate risk-taking, the more the enterprise tends to invest in high-risk, high net present value projects (Chong et al., 2018). As high-risk projects tend to generate higher expected returns than low-risk projects, a reasonable level of corporate risk-taking is an important reference for maintaining long-term competitiveness and increasing the long-term value of enterprises. A considerable amount of research has shown that the level of corporate risk-taking is one of the fundamental forces that drive long-term and high-quality economic growth (Boubakri et al., 2013; Faccio et al., 2016). From the microcosmic view, higher risk-taking helps enterprises obtain more profits and wealth, maintain long-term competitive advantage, and improve the value and capital allocation efficiency of enterprises (Li et al., 2021). From the macroscopic view, corporate risk-taking is conducive to promoting technological progress, accelerating social capital accumulation, upgrading industrial structure and improving social productivity (Habib and Hasan, 2017). However, some scholars have attributed the root causes of the United States financial crisis to excessive risk-taking, arguing that excessive risk-taking can lead to more serious economic consequences (Xiaorong and Ruijun, 2014).

As an important informal institutional arrangement, the network relationships embedded in social networks have built a channel for enterprises to share scarce resources and exchange heterogeneous information. In recent years, research on social networks has received increasing attention in the field of organizational psychology. Traditional organizational psychology research has focused on the attributes of actors in organizations in isolation, i.e., the capabilities and characteristics of actors. In contrast, contemporary scholars focus on the relationships among actors in organizations, i.e., how actors use relational network opportunities to gain appropriate social capital, which in turn ultimately influences the organization’s own behavior and decisions (Brass, 2012). Corporate risk-taking has a strong resource dependence, which is not only influenced by the subjective willingness of decision-makers to take risks, but also by the objective limitations on the enterprise’s ability to access resources (Ferris et al., 2019). In addition, high-risk projects often require more start-up capital, and the ability of enterprises to access external resources and the level of financing constraints they face can affect the attitude of their managers toward risk. Relevant psychological studies point out that social capital plays an important role in influencing the behavior and decisions of firms (Lazarova and Taylor, 2009). Given that social capital embedded in social networks can provide the necessary resources to support corporate risk-taking, this paper focuses on the impact of social networks in the form of interlocking directors on corporate risk-taking by studying the following questions: (i) Can NET significantly improve RISK? (ii) Through what channels does NET play a role in RISK? (iii) Is there any difference in the impact of NET on RISK for enterprises with different ownership nature and industry competition intensity?

In order to answer the above questions, based on the network embeddedness perspective and using the data of Chinese A-share listed firms from 2007 to 2020 as a sample to construct the interlocking director network of listed companies, in this study, we systematically examine the influence of network centrality indicators and structural hole richness of NET on RISK. It is found that NET can indeed significantly enhance RISK, and the higher the network centrality and the richer the structural holes, the higher the level of RISK. Financing constraints and R&D investment play a mediating role in the process of NET influencing RISK. The NET can enhance RISK: by alleviating financing constraints and increasing R&D investment. Further subdividing the nature of corporate ownership and the intensity of industry competition, it is found that the promotion effect of NET on RISK is more significant in non-state-owned firms and firms with higher industry competition intensity.

Compared with the existing literature, the main innovations and contributions of this paper mainly lie in three aspects. First, it expands the research perspective on the economic consequences of interlocking director network and the influencing factors of corporate risk-taking. Current research has focused on the influence of interlocking director network on enterprise innovation (Chuluun et al., 2017; Jiang et al., 2020), enterprise strategic decision-making (Deutsch et al., 2011; Zou et al., 2019), enterprise value (Larcker et al., 2013; Zona et al., 2018) and corporate social responsibility (Xiaoqing et al., 2020), and less attention has been paid to the important role played by interlocking director network, an informal social network relationship, in corporate risk-taking. In addition, most of the literature on the influencing factors of corporate risk-taking is based on the principal-agent theory framework, and scholars have studied the impact of enterprise characteristics (Peltomäki et al., 2021), corporate governance (Nakano and Nguyen, 2012; Sila et al., 2016; Gopalan et al., 2021), managers’ characteristics (Zhu and Chen, 2015; Ferris et al., 2019) and relevant systems and policies (Li et al., 2013; Langenmayr and Lester, 2018) on corporate risk-taking from the micro-level and external macro-environment. This paper, however, takes a new perspective of social network embeddedness and uses network centrality and structural hole richness indicators to study the impact of NET on RISK, extending the research perspective on the influencing factors of corporate risk-taking. Second, compared with Su and Liu’s study, this paper further analyses and tests the influence mechanism of NET on RISK by introducing two mediating variables, namely financing constraints and R&D investment, which makes the influence channel of NET on RISK clearer and more complete, and provides some theoretical reference and empirical basis for promoting the improvement of corporate risk-taking under the background of economic transformation (Su and Liu, 2019). Third, the ability of enterprises to access resources varies across different nature and competitive environments. Therefore, based on Su and Liu’s research, this paper further reveals the boundary conditions of the influence of NET on RISK from the perspective of the nature of the ownership and the intensity of industry competition, which is helpful for enterprises to take appropriate risk investment behavior in the face of the complex and changeable market competition environment.

## 2. Theory and hypothesis

### 2.1. Interlocking director network and corporate risk-taking

According to resource dependency theory, an important element of corporate risk-taking is access to abundant scarce resources and critical information (Zona et al., 2018). Previous studies have shown that the social network of enterprises is beneficial for enterprises to obtain relevant knowledge, experience and information resources required for risk-taking behavior (Dbouk et al., 2020). As an important social network relationship, the interlocking director network is a direct or indirect inter-enterprise network established by directors who serve on the boards of two or more enterprises at the same time through cross-servicing (Bianchi et al., 2020). At present, most scholars describe the position of enterprises in the network from two aspects: centrality and structural holes. “Centrality” mainly describes whether an enterprise is near the center or the edge of the network. The “structural hole” is a bridge for two enterprises that have no direct connection. Because the position of enterprises in the network is an important factor determining their ability to obtain resources, this study mainly uses centrality and structural holes to explain the impact of NET on RISK. Interlocking directors establish a connection between the internal organization and the external market environment by serving on the boards of multiple corporations, which provides enterprises with rich heterogeneous resources and an important channel for information sharing (Deren and Yunsen, 2012). Based on the existing literature, the influence of NET on RISK is mainly reflected in the governance effect and resource effect.

In terms of governance effect, the existence of agency problems will reduce the level of RISK. For the sake of self-interest, managers are more likely to adopt a relatively stable investment strategy to avoid personal wealth loss, dismissal risk and professional reputation loss caused by investment failure (John et al., 2008). The interlocking director network can influence the ability of corporate risk-taking through the function of the board of directors. On the one hand, interlocking directors can use their central position in the network to gain more information, resources and knowledge of governance behavior, which can enhance their decision-making influence on the board of directors and accumulate more personal reputation capital for them (Larcker et al., 2013). This reputation capital will strengthen their supervision effect and make them more motivated and stricter to restrain and supervise the self-interest and rent-seeking behavior of managers, which further eases the agency conflict of enterprises. When the agency conflict is alleviated, enterprise managers are more willing to invest in venture capital projects that contribute to corporate growth and have a positive net present value to enhance the level of RISK. On the other hand, interlocking directors at the center of the network and occupying the position of structural holes can provide enterprises with more abundant and diversified information resources. These heterogeneous information resources are conducive to enhancing directors’ right to advise on company management decisions, and help managers make more informed risk investment decisions (Chao and Jianjun, 2018). Relevant psychological research shows that enterprises can gain social capital such as knowledge and experience by observing and emulating the superior strategic behavior of partners in the network, which helps them make better decisions (Xie et al., 2020; Tian et al., 2021).

In terms of resource effect, risk-taking is a resource-consuming activity (Ferris et al., 2019). The social capital and information channels brought by the interlocking director network can alleviate the dependence of corporate risk-taking behavior on external resources and help improve the level of risk-taking. First, as an informal institutional arrangement, the interlocking director network has the advantages of low connection cost, stable connection and effective connection, which can help companies obtain more resources at a lower cost (Larcker et al., 2013). Specifically, the higher the centrality of the interlocking director network of a company, the more relationships it establishes with other companies, the shorter the transmission path of information and resources, and the faster the company has access to core resources and effective information. In addition, enterprises occupying structural holes function as information dissemination “bridges” in the network, which can connect enterprises that are not directly connected to access heterogeneous resources and key information needed for risk-taking behavior (Tortoriello, 2015). Second, the root of the resource constraint problem faced by enterprises lies in the information asymmetry among enterprises. Enterprise relationship network embedding can accelerate the rapid transmission and flow of information among enterprises, increase the frequency of information communication and resource-sharing opportunities, reduce the information asymmetry in the investment process and create more venture capital projects and strategic implementation platforms for enterprises. Accordingly, Hypothesis 1 is proposed:

*H1*: Interlocking director network is positively related to corporate risk-taking. The higher the centrality or the richer the structural holes, the higher the level of risk-taking.

### 2.2. The mediating effect of financing constraints

As an important factor restricting the development of enterprises, financing constraints have been the focus of academic research. Studies have shown that the interlocking director network can alleviate the financing constraints of enterprises through the advantage of network location (Xiaoqing et al., 2020). For one thing, the embedding of the interlocking director network relationship provides information channels for enterprises to communicate with the outside world, which can alleviate the information asymmetry between enterprises and fund providers, help enterprises obtain more external funds and financing channels at a lower cost, and reduce the difficulty of external financing. Wang et al. (Ying and Tingqiu, 2014) found the embedding of the director network is beneficial to increase the ability of enterprises to obtain debt financing. The higher the network centrality of the enterprise, the more access to debt resources and information, and the lower the cost of debt financing. The work of Chuluun et al. (2014) suggested that the information transmission function of the director network is beneficial in reducing the financing constraints arising from information asymmetry between the enterprise and external creditors. For another, the interlocking director network can also affect the efficiency of corporate governance. Gertler (1989) pointed out that agency problems will affect the level of corporate financing constraints. The social capital and reputation capital brought by the interlocking director network to the enterprise can reduce management’s agency problems, improve corporate governance efficiency and alleviate the financing constraints of the enterprise.

The venture capital projects of enterprises are characterized by long investment cycles, large capital investments and many uncertainties, which means that enterprises need sufficient resources to support the investment process. However, the financing constraints faced by enterprises in the investment process will restrict the ability of enterprises to obtain resources and reduce the level of corporate risk-taking. Yan et al. (Ruosen et al., 2020) found that enterprises with higher financing constraints will tend to avoid high-risk investment projects in order to increase the success rate of loans, which will reduce corporate risk-taking. However, enterprises can use their location advantage in the social network to access resources and alleviate financing constraints. Enterprises with higher network centrality and richer structural holes will have a stronger ability to access information and resources (Xiaoqing et al., 2020). Therefore, the interlocking director network of enterprises can help enterprises obtain the resources required for venture capital investment at a lower financing cost and improve the level of corporate risk-taking. On this basis, Hypothesis 2 is proposed:

*H2*: Financing constraints play a mediating role in the relationship between NET and RISK. The NET can improve RISK by reducing financing constraints.

### 2.3. The mediating effect of R&D investment

Corporate R&D activities are a high-risk, high-return strategic behavior with high requirements for innovative resources and information. Resource allocation and R&D decisions of enterprises are often decided by the board of directors, in which interlocking directors play an important role in this process. Interlocking directors are a reliable and low-cost network of inter-enterprise relationships where enterprises can exchange resources and share information to obtain the resources and information flow needed for R&D activities (Jiang et al., 2020). Network centrality and structural holes are key indicators to measure the network position of interlocking directors. Compared with enterprises at the edge of the network, enterprises occupying the central position of the network are more likely to obtain new information and new resources related to R&D activities (Ying and guangli, 2018). These new information and resources help to promote technology exchange among enterprises, reduce the cost of trial-and-error and investment risks of enterprise innovation and R&D, and increase the motivation of enterprise R&D investment. Generally speaking, enterprises in the structural hole position possess a large amount of heterogeneous information and resources, which can be integrated and utilized to increase the motivation for enterprises to invest in R&D.

The R&D investment of enterprises is strongly related to the level of risk-taking (Dewett, 2007). In general, enterprises with more R & D investment have a stronger risk appetite. Banerjee and Gupta (2019) found that the R&D investment of enterprises will significantly promote the level of risk-taking. Therefore, it is important for enterprises to appropriately increase their risk-taking level once they are involved in investment decisions related to R&D projects. However, the R&D investment of enterprises is an investment with high risk, long cycle, and uncertain return. Only when enterprises obtain new resources and information from the outside for a long time can they ensure the smooth progress of R&D projects (Ying and guangli, 2018). The key technical resources and rich technical experience brought by the interlocking director network are conducive to prompting managers to increase the R&D investment intensity of enterprise innovation activities and enhance the level of risk-taking. Therefore, hypothesis 3 is proposed:

*H3*: R&D investment plays a mediating role in the relationship between NET and RISK. The NET can improve RISK by increasing corporate R&D investment.

**Figure 1:**
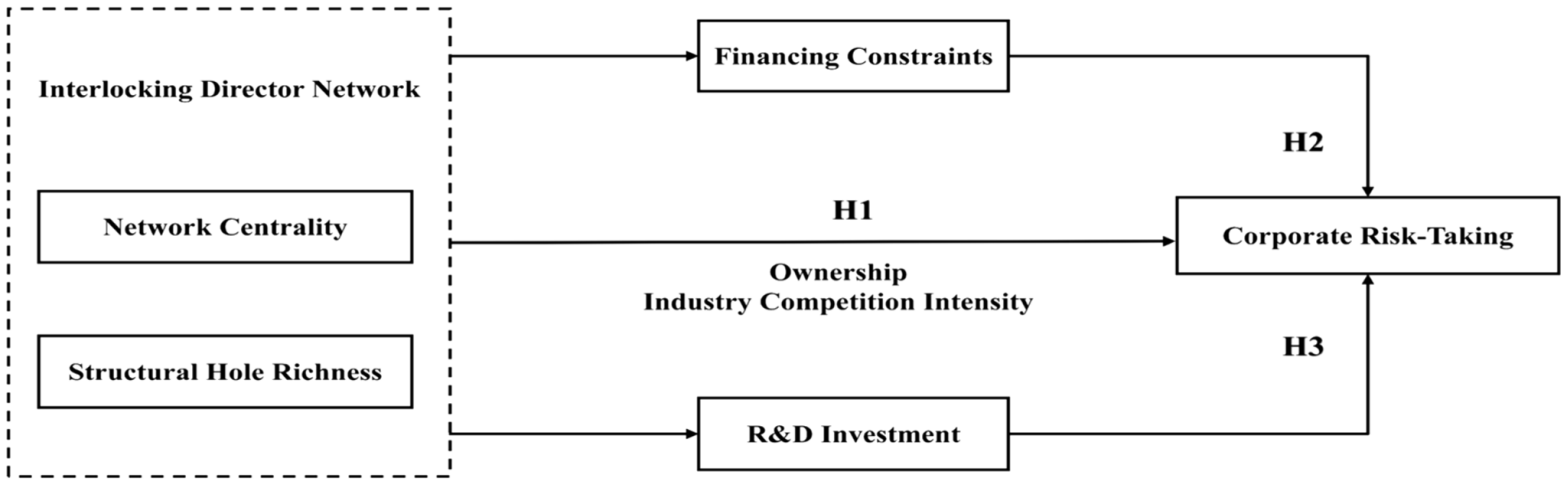
Research framework.

In summary, the overall research framework of this paper is shown in Figure 1.

## 3. Data and research method

### 3.1. Sample and data

Considering that the China Securities Regulatory Commission regulated the R&D expenditure of listed companies in 2007, we selected the data of Chinese A-listed companies from 2007 to 2020 as the research sample. In order to prevent the interference of heterogeneous factors, the sample data were processed as follows: (1) excluding the sample of companies in the financial and insurance industries; (2) excluding ST, PT and delisted samples; (3) excluding the sample with missing data concerning financial or governance; and (4) considering the influence of abnormal values, the main continuous variables were tailed (winsorize) at the upper and lower 1% levels. Finally, a total of 19,689 annual-firm data were obtained. The data of corporate risk-taking and R&D investment in this study were obtained from the WIND database, the data of the interlocking director network and other listed companies’ finance and governance were obtained from the CSMAR database, and the data of GDP growth rate was from China Statistical Yearbook. In order to ensure the accuracy and completeness of the data to the greatest extent, the CSMAR database was used to check and supplement the R&D investment data.

Regarding the data related to the NET, this study first obtained the employment data of directors of listed companies from the personal characteristic files in the CSMAR database, and excluded the samples of non-directors who held positions in other listed companies. Next, we used the programming function of PYTHON software to convert the “2-mode” matrix of “director-company” into the “1-mode” matrix of “company-company.” If two companies have the same director, the elements of the matrix are recorded as 1, or else 0. Then, the matrix of each year was imported into UCINET and converted into a net file that could be recognized by PAJEK. Finally, we used PAJEK software to calculate the network centrality and structural hole indicators by year.

### 3.2. Variable definition

#### 3.2.1. Dependent variable

Based on the studies of Faccio et al. (2016) and Li et al. (Wenggui and Minggui, 2012), we used the volatility of corporate surplus to measure the level of RISK. Since executive tenure in Chinese listed companies is typically 3 years, this study used every 3 years (*t*–2, *t*) as an observation period. To eliminate the effects of industry and economic cycles, we calculated the standard deviation (RISK1) and extreme deviation (RISK2) of return on assets adjusted by the industry and annual average to measure RISK, respectively. In addition, because of the large number of manufacturing companies in China, we refined the industry categorization of manufacturing companies to secondary codes and removed the sample of only one company in the industry. The specific calculation process of corporate risk-taking can be seen in the following equation:


(1) $$\mathrm{Adj}\_{\mathrm{Roa}}_{i,t}=\frac{{\mathrm{EBIT}}_{i,t}}{{\mathrm{ASSET}}_{i,t}}-\frac{1}{X}\overset{X}{\underset{k=1}{\sum }}\frac{{\mathrm{EBIT}}_{k,t}}{{\mathrm{ASSET}}_{k,t}}.$$

(2) $$\mathrm{RISK}1_{i,t}=\sqrt{\frac{1}{T-1}\overset{T}{\underset{t=1}{\sum }}{\left(\mathrm{Adj}\_{\mathrm{Roa}}_{i,t}-\frac{1}{T}\overset{T}{\underset{t=1}{\sum }}\mathrm{Adj}\_{\mathrm{Roa}}_{i,t}\right)}^{2}}|T=3$$

(3) $$\mathrm{RISK}2_{i,t}=\max\left(\mathrm{Adj}\_{\mathrm{Roa}}_{i,t}\right)-\min\left(\mathrm{Adj}\_{\mathrm{Roa}}_{i,t}\right)$$

where $\mathrm{Adj}\_{\mathrm{Roa}}_{i,t}$ represents the return on assets adjusted by the industry and annual average; EBIT is the profit before interest and tax; ASSET is the total assets at the end of the year; the subscripts i indicates the company and t indicates the year; X and k respectively represent the total number of enterprises in a certain industry and the *k*-th enterprise in the industry; T=3 represents a 3-year observation period.

#### 3.2.2. Independent variables

The independent variables in this study are the centrality and structural holes of NET. Network centrality focuses on the self-directed connected nature of a company, and to some extent reflects the importance and influence of that company in the overall network. Previous studies mostly used three indicators: degree centrality, closeness centrality and betweenness centrality. However, the closeness centrality has higher requirements on the network, and it can only be done when the network is completely connected, so this indicator is rarely used. Therefore, drawing on the methods of Xie and Chen (Deren and Yunsen, 2012), we adopted two indicators, degree centrality (Degree) and betweenness centrality (Betweenness), to measure the centrality of interlocking directors of listed companies in the overall network. The calculation formula is shown below:


(4) $${\mathrm{Degree}}_{i}=\frac{\sum_{j\neq i}X_{ij}}{g-1}$$

where i is the focal company and j is other companies other than i in that year; $X_{ij}$ is a network connection, if company i and j have at least one interlocking director, the $X_{ij}=1$ , or else $X_{ij}=0$ ; g is the total number of companies in the network, and due to the year difference, (g−1) is used in this paper to eliminate the effect of the network size difference.


(5) $${\mathrm{Betweenness}}_{i}=\frac{\underset{j<k}{\sum }g_{jk\left(n_{i}\right)}/g_{jk}}{\left(g-1\right)\left(g-2\right)/2}$$

where $g_{jk}$ is the number of shortest paths between company j and k ; $g_{jk\left(n_{i}\right)}$ is the number of shortest paths between company j and k , and through company i ; g is the number of companies in the interlocking director network, and (g−1)(g−2)/2 is used to eliminate the effect of the network size difference of listed companies.

**Figure 2:**
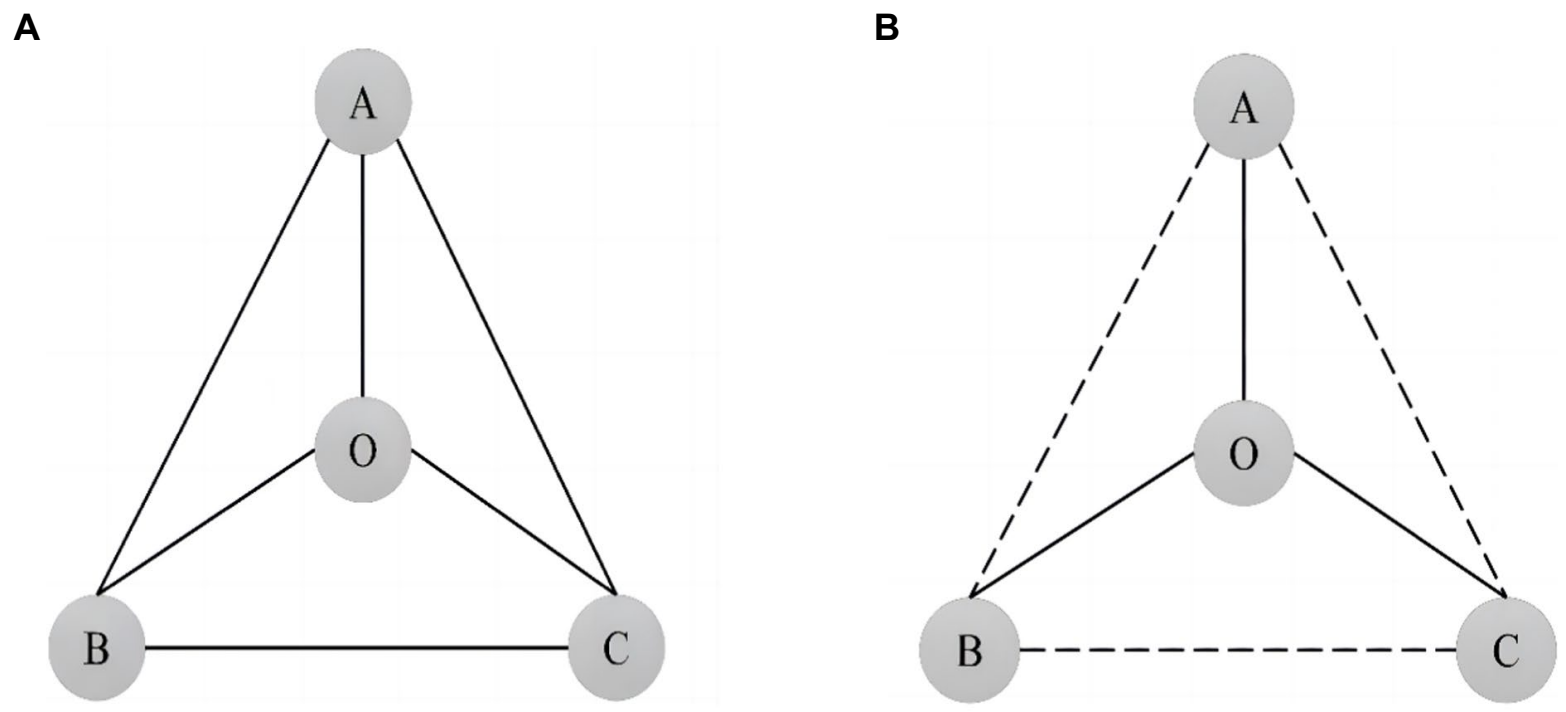
Schematic diagram of structural holes. **(A)** Information flow network without structural holes. **(B)** Information flow network with structural holes.

Different from network centrality, structural holes focus more on the non-redundant connections between two actors. As shown in Figure 2A, four independent individual actors A, B, C, and O have direct connections (represented by solid lines) between them, and each actor has the same positional advantage in the network and the same access to resources and information, so there is no structural hole in this network. However, in contrast, see Figure 2B, there is no direct connection between the three actors A, B, and C (indicated by dashed lines), but there is a direct connection between actor O and A, B, and C. At this time, O becomes the only channel for communication among A, B, and C. The flow direction of information and resources in the network is controlled by O, and O occupies the structural hole position in the whole network.

The calculation methods of the structural hole index include effective size, efficiency, constraint and hierarchy, among which the constraint index is more widely used. Following the study of Tortoriello (2015), this paper measured the richness of the structural holes (SH) of the interlocking director network by the difference between 1 and Constraint. SH is calculated as follows:


(6) $${\mathrm{SH}}_{i}=1-{\left(P_{ij}+\underset{q}{\sum }P_{iq}P_{qj}\right)}^{2},q\neq i,j$$

where $P_{ij}$ represents the direct connection strength between company i and company j ; $\underset{q}{\sum }P_{iq}P_{qj}$ is the sum of the strength of the indirect connection between company i and company j where passing through company q is the only way; ${\left(P_{ij}+\underset{q}{\sum }P_{iq}P_{qj}\right)}^{2}$ represents the degree to which company i is constrained by company j . The larger the SH, the richer the structure holes of the interlocking director network.

#### 3.2.3. Control variables

Based on the studies of Zhang et al. (Min et al., 2015) and Boubakri et al. (2013), this study selected firm size (Size), leverage level (Lev), firm age (Age), shareholding proportion of the largest shareholder (Top1), profitability (ROA), firm growth (Growth), capital expenditure level (Cap) and GDP growth rate (Gdp) as control variables. The details are reported in Table 1.

**Table 1:** Definition and description of main variables.

Type	Name	Code	Definition
Dependent variable	Corporate risk-taking	RISK1	Standard deviation calculated from Equation (2)
		RISK2	The extreme deviation calculated from Equation (3)
Independent variables	Degree centrality	Degree	See Equation (4), calculated by PAJEK software
	Betweenness centrality	Betweenness	See Equation (5), calculated by PAJEK software
	Structural hole richness	SH	See Equation (6), calculated by PAJEK software
Control variables	Firm size	Size	Natural logarithm of total assets at the end of the year
	Leverage level	Lev	Total liabilities/total assets
	Firm Age	Age	Natural logarithmic of listing age plus one
	Ownership concentration	Top1	Shareholding proportion of the largest shareholder at the end of the year
	Profitability	ROA	Net profit/total assets
	Firm growth	Growth	Sales revenue growth rate
	Capital expenditure level	Cap	Capital expenditure/total assets
	GDP growth rate	Gdp	GDP growth rate, data from China Statistical Yearbook

### 3.3. Model setting

In order to test the effect of NET on RISK, the following regression model was constructed to test hypothesis H1:


(7) $$\begin{array}{c} RISK_{i,t}=\alpha_{0}+\alpha_{1}NET_{i,t}+\alpha_{2}Controls_{i,t}\\ +\sum Company_{i}+\sum Year_{t}+\varepsilon_{i,t} \end{array}$$

where *i* and *t* represent company and year respectively; *NET* denotes the relevant measurement indicators of interlocking director network, specifically including Degree, Betweenness and SH; Controls represents all control variables in Table 1; *Company* and Year respectively represents individual and year fixed effects; ε is the error term that is assumed to be normally distributed with zero mean value and constant variance (Elahi et al., 2021). This paper used the two-way fixed effects model of panel data to estimate, and further conducted clustering processing at the company level.

In order to further explore the influence mechanism of NET on RISK, this paper introduces two mediating variables, financing constraint and R&D investment, and drawing on the application of the mediating effect test by Wen et al. (Zhonglin et al., 2004), the following mediating effect model is constructed on the basis of Equation (7) to test hypothesis 2 and hypothesis 3:


(8) $$\begin{array}{c} SA/R\&D_{i,t}=\gamma_{0}+\gamma_{1}NET_{i,t}+\gamma_{2}Controls_{i,t}\\ +\sum Company_{i}+\sum Year_{t}+\varepsilon_{i,t} \end{array}$$

(9) $$\begin{array}{c} RISK_{i,t}=\beta_{0}+\beta_{1}NET_{i,t}+\beta_{2}SA/R\&D_{i,t}\\ +\beta_{3}Controls_{i,t}+\sum Company_{i}+\sum Year_{t}+\varepsilon_{i,t} \end{array}$$

where *SA* indicates the measure of financing constraint, and drawing on the method of Li et al. (Xiaoqing et al., 2020), the absolute number of the SA index, which is constructed based on the two variables of Size and Age with little change over time and high exogeneity, is used to calculate the level of financing constraint, and the SA index is constructed in the way shown in Equation (10). The larger the absolute number of SA index, the more serious the degree of financing constraint of the enterprise; *R* & *D* indicates the measure of corporate R&D investment, and this paper adopts the relative indicator-R&D investment intensity, the proportion of R&D expenditure to operating revenue, by referring to Yan et al.’s research (Ruosen et al., 2020).


(10) $$SA=-0.737Size+0.043Size^{2}-0.040Age$$

## 4. Results and discussion

### 4.1. Descriptive statistics

Table 2 represents the results of descriptive statistics for the main variables. The mean value (standard deviation) of RISK1 and RISK2 is 0.0321 (0.0428) and 0.0606 (0.0796) respectively. The standard deviations of both are greater than the mean, indicating that the level of corporate risk-taking among different companies is quite different. The mean (median) of Degree and Betweenness is 0.0013 (0.0011) and 0.0016 (0.0008) respectively, and the maximum (minimum) value is 0.0043 (0.0002) and 0.0114 (0.0000) respectively, indicating that although most listed companies in China have established interlocking director network, the degree of network connection varies greatly. In addition, the mean value of SH is 0.5354, which is much larger than the mean value of the network centrality index, and the difference between the maximum value and the minimum value is 0.9110, which indicates that compared with the network centrality indicator, the structural hole richness of interlocking director network varies more significantly between listed companies.

**Table 2:** Descriptive statistics of main variables.

Name	Code	*N*	Mean	SD	Median	Min	Max
Corporate risk-taking	RISK1	19,689	0.0321	0.0428	0.0189	0.0001	0.4565
	RISK2	19,689	0.0606	0.0796	0.0361	0.0001	0.9065
Degree centrality	Degree	19,689	0.0013	0.0008	0.0011	0.0002	0.0043
Betweenness centrality	Betweenness	19,689	0.0016	0.0022	0.0008	0.0000	0.0114
Structural hole richness	SH	19,689	0.5354	0.2393	0.6039	0.0000	0.9110
Firm size	Size	19,689	22.2491	1.2707	22.0707	19.9096	26.2497
Leverage level	Lev	19,689	0.4374	0.1980	0.4323	0.0624	0.8927
firm age	Age	19,689	2.2529	0.6445	2.3026	1.0986	3.2958
Ownership concentration	Top1	19,689	0.3411	0.1445	0.3196	0.0903	0.7349
Profitability	ROA	19,689	0.0338	0.0664	0.0341	−0.2918	0.2006
Firm growth	Growth	19,689	0.1718	0.4166	0.1056	−0.5109	2.7446
Capital expenditure level	Cap	19,689	0.0484	0.0438	0.0354	0.0008	0.2137
GDP growth rate	Gdp	19,689	6.7699	2.2185	6.9500	2.2400	14.2300

### 4.2. Correlation analysis

Table 3 presents the Pearson correlation coefficient matrix of the main variables. It can be seen that the four indicators of interlocking director networks (Degree, Betweenness and SH) are significantly and positively correlated (*p* < 0.01) with the level of corporate risk-taking (RISK1 and RISK2), which is consistent with the prediction of H1 in this paper. The significant results of control variables and RISK are generally consistent with the findings of Zhang et al. (Min et al., 2015) and Faccio et al. (2016). In addition, the correlation coefficient between the control variables is less than 0.5, which indicates that the possibility of collinearity in the model is low.

**Table 3:** Pearson correlation coefficient matrix.

	RISK1	RISK2	Degree	Betweenness	SH	Size	Lev	Age	Top1	ROA
RISK1	1									
RISK2	0.998^***^	1								
Degree	0.072^***^	0.071^***^	1							
Betweenness	0.050^***^	0.050^***^	0.800^***^	1						
SH	0.036^***^	0.036^***^	0.733^***^	0.637^***^	1					
Size	−0.161^***^	−0.163^***^	0.103^***^	0.190^***^	0.182^***^	1				
Lev	0.057^***^	0.056^***^	0.097^***^	0.092^***^	0.075^***^	0.453^***^	1			
Age	0.084^***^	0.084^***^	0.072^***^	0.131^***^	0.126^***^	0.414^***^	0.323^***^	1		
Top1	−0.134^***^	−0.133^***^	0.053^***^	0.032^***^	0.015^**^	0.231^***^	0.072^***^	−0.049^***^	1	
ROA	−0.392^***^	−0.386^***^	0.058^***^	0.043^***^	0.021^***^	0.038^***^	−0.329^***^	−0.130^***^	0.128^***^	1
Growth	0.039^***^	0.038^***^	0.027^***^	0.004	0.010	0.036^***^	0.021^***^	−0.077^***^	0.0100	0.229^***^
Cap	−0.087^***^	−0.087^***^	0.055^***^	0.026^***^	−0.010	−0.002	−0.037^***^	−0.218^***^	0.036^***^	0.138^***^
Gdp	−0.108^***^	−0.108^***^	0.321^***^	0.121^***^	0.005	−0.137^***^	0.050^***^	−0.089^***^	0.079^***^	0.066^***^

^*^p < 0.1, ^**^p < 0.05, ^***^p < 0.01.

### 4.3. Interlocking director network characteristics

**Figure 3:**
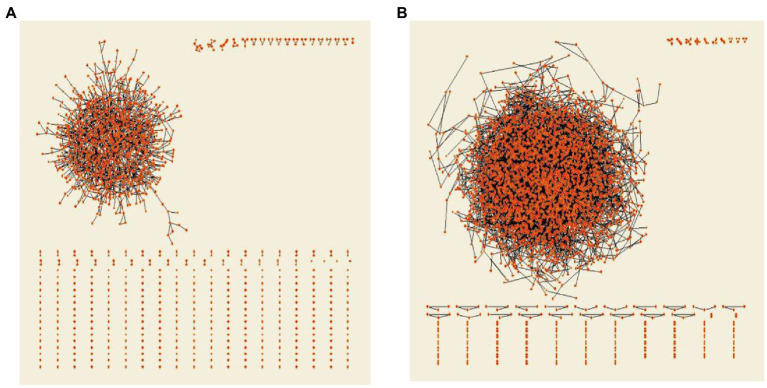
Comparison of interlocking director network of listed companies in 2007 and 2020. **(A)** Interlocking director network of Chinese A-listed companies in 2007. **(B)** Interlocking director network of Chinese A-listed companies in 2020.

In order to describe the distribution structure and aggregation degree of the interlocking director network of Chinese listed companies more intuitively, as well as the dynamic changes of the interlocking director network over time, this paper used PAJEK software to visualize the interlocking director network of Chinese listed companies in 2007 (A) and 2020 (B) respectively, as shown in Figure 3. Observing Figure 3A, it can be found that in 2007, the interlocking director network of Chinese listed companies has already reached a certain scale, and most companies have entered the largest connected sub-network in the network, but there are still many companies in a free state. Continuing to observe Figure 3B, it can be seen that in 2020, the scale of the largest connected sub-network of the interlocking director network of Chinese listed companies has further expanded significantly, and there are few isolated nodes, which indicates that the structure of the interlocking director network of Chinese listed companies has become more complex and closer as time goes on.

### 4.4. Baseline regression results

Table 4 reports the baseline regression results of the impact of NET on RISK. Columns (1)–(4) use the network centrality indicator, and columns (5) and (6) use the structural hole richness indicator. Table 4 shows that the interlocking director network centrality indicator (Degree, Betweenness and SH) positively affects corporate risk-taking (RISK1 and RISK2) at the 1% significance level, and the structural hole richness positively affects corporate risk-taking at the 5% level, which indicates that the higher the network centrality and the richer the structural hole, the higher the level of corporate risk-taking. Accordingly, H1 is supported.

**Table 4:** Interlocking director network and corporate risk-taking.

Name	Code	(1)	(2)	(3)	(4)	(5)	(6)
		RISK1	RISK2	RISK1	RISK2	RISK1	RISK2
Degree centrality	Degree	1.432^***^	2.713^***^				
		(2.76)	(2.78)				
Betweenness centrality	Betweenness			0.497^***^	0.929^***^		
				(2.87)	(2.87)		
Structural hole richness	SH					0.004^**^	0.008^**^
						(2.50)	(2.51)
Firm size	Size	−0.020^***^	−0.038^***^	−0.021^***^	−0.039^***^	−0.020^***^	−0.039^***^
		(−11.48)	(−11.46)	(−11.51)	(−11.49)	(−11.49)	(−11.47)
Leverage level	Lev	0.038^***^	0.071^***^	0.038^***^	0.072^***^	0.038^***^	0.071^***^
		(5.51)	(5.58)	(5.52)	(5.58)	(5.50)	(5.57)
Firm age	Age	0.017^***^	0.033^***^	0.018^***^	0.033^***^	0.018^***^	0.033^***^
		(5.85)	(5.87)	(5.87)	(5.88)	(5.88)	(5.90)
Ownership concentration	Top1	−0.013	−0.024	−0.013	−0.024	−0.013	−0.023
		(−1.44)	(−1.36)	(−1.44)	(−1.36)	(−1.42)	(−1.35)
Profitability	ROA	−0.213^***^	−0.384^***^	−0.213^***^	−0.385^***^	−0.213^***^	−0.384^***^
		(−17.95)	(−17.50)	(−17.97)	(−17.52)	(−17.94)	(−17.49)
Firm growth	Growth	0.006^***^	0.011^***^	0.006^***^	0.011^***^	0.006^***^	0.011^***^
		(5.30)	(5.26)	(5.32)	(5.28)	(5.29)	(5.25)
Capital expenditure level	Cap	−0.011	−0.025	−0.011	−0.025	−0.011	−0.024
		(−1.08)	(−1.28)	(−1.09)	(−1.30)	(−1.05)	(−1.26)
GDP growth rate	Gdp	−0.001^***^	−0.002^***^	−0.001^***^	−0.002^**^	−0.001^**^	−0.002^**^
		(−2.81)	(−2.77)	(−2.62)	(−2.58)	(−2.51)	(−2.47)
Constant	Cons	0.459^***^	0.862^***^	0.460^***^	0.865^***^	0.458^***^	0.860^***^
		(11.51)	(11.49)	(11.54)	(11.52)	(11.50)	(11.47)
Company FE	Company	Yes	Yes	Yes	Yes	Yes	Yes
Year FE	Year	Yes	Yes	Yes	Yes	Yes	Yes
Observations	N	19,689	19,689	19,689	19,689	19,689	19,689
Goodness of fit	Adj. R^2^	0.249	0.246	0.249	0.246	0.249	0.246
*F*-value	F	38.81^***^	39.36^***^	38.76^***^	39.31^***^	38.74^***^	39.30^***^

^*^p < 0.1, ^**^p < 0.05, ^***^p < 0.01. *T*-statistics are listed in parentheses, which have been clustered at the company level, and the following table is the same. Estimates of control variables are omitted from subsequent tests due to space constraints.

The regression results for the control variables in Table 4 are generally consistent with studies of Li et al. (Wenggui and Minggui, 2012) and Zhang et al. (Min et al., 2015): the coefficient of Size is significantly negative, indicating that small firms have a stronger risk appetite; the estimated coefficient of Lev is significantly positive, indicating that the higher the level of debt, the higher the level of risk-taking; the regression coefficient of Age is significantly positive, indicating that the longer the firm has been listed, the higher the level of risk-taking; the coefficient of ROA is significantly negative, indicating that the less profitable the firm is, the more it wants to improve its profitability through risk-taking behavior; the regression coefficient of Growth is significantly positive, indicating that the more growth opportunities the firm has, the more it tends to increase its level of RISK in order to make full use of investment opportunities; the regression coefficient of Gdp is obviously negative, indicating that overheating economy will reduce the risk tolerance of firms.

### 4.5. Robustness tests

#### 4.5.1. Endogeneity test

Although the panel data fixed effects model used in this study can control for partial omitted variable bias, the above regression analysis may also suffer from the endogeneity problem due to reverse causality. Enterprises with higher risk-taking level may be more likely to attract interlocking directors who are at higher network centrality and occupy more structural holes to serve. Therefore, in order to mitigate the potential endogeneity problem, we adopted the two-stage least squares (2SLS) method to re-examine the baseline regression results. Based on Wang et al. (Yongqing et al., 2019), we chose one-period lagged indicators of network centrality and structural holes as instrumental variables (IV), and the results are shown in Table 5. In the first stage, the coefficients of the IV are significantly positive, and the K-P rk LM statistic rejects the hypothesis of “under-identification of instrumental variables” at the 1% level, indicating that there is no under-identification problem. The C-D Wald F and K-P rk Wald F statistics for testing weak instrumental variables are both much larger than the critical value of 16.38 at the 10% significance level, indicating that there is no problem of weak instrumental variables. In addition, since the number of selected instrumental variables is exactly equal to the number of endogenous variables, there is no over-identification problem. In summary, the selected instrumental variables are valid. In the second stage, the regression results in columns (1)–(6) show that the network centrality (Degree and Betweenness) and structural holes (SH) are significantly and positively correlated with RISK, which indicates that the contribution of NET to RISK still holds after controlling for possible endogeneity.

**Table 5:** Regression results of the two-stage least squares (2SLS).

Name	Code	(1)	(2)	(3)	(4)	(5)	(6)
		RISK1	RISK2	RISK1	RISK2	RISK1	RISK2
Degree centrality	Degree	3.479^***^	6.482^***^				
		(2.94)	(2.95)				
Betweenness centrality	Betweenness			1.303^***^	2.441^***^		
				(2.88)	(2.90)		
Structural hole richness	SH					0.013^**^	0.025^**^
						(2.44)	(2.51)
Constant	Cons	0.438^***^	0.822^***^	0.443^***^	0.832^***^	0.434^***^	0.816^***^
		(9.67)	(9.72)	(9.71)	(9.76)	(9.67)	(9.72)
Control variables	Controls	Yes	Yes	Yes	Yes	Yes	Yes
Company FE	Company	Yes	Yes	Yes	Yes	Yes	Yes
Year FE	Year	Yes	Yes	Yes	Yes	Yes	Yes
Observations	*N*	15,857	15,857	15,857	15,857	15,857	15,857
Goodness of fit	Adj. *R*^2^	0.269	0.267	0.268	0.266	0.269	0.266
Results of the first stage	The first stage	0.449^***^		0.400^***^		0.349^***^	
Underidentification test	K-P rk LM statistic	1290.159^***^		645.574^***^		858.418^***^	
Weak identification test	C-D Wald *F* statistic	3782.805^***^		2672.932^***^		1832.166^***^	
	K-P rk Wald *F* statistic	2077.445^***^		935.649^***^		1081.699^***^	

K-P rk LM, Kleibergen-Paap rk LM statistic. C-D Wald *F*, Cragg-Donald Wald *F* statistic. K-P rk Wald F, Kleibergen-Paap rk Wald *F* statistic. *p<0.1, **p<0.05, ***p<0.01.

Although the endogeneity problem due to reverse causality could be controlled to a certain extent by adopting the 2SLS method, in order to avoid possible non-random interference of NET affecting RISK, this study further adopted the propensity score matching method (PSM) to further mitigate the endogeneity problem due to sample selection bias. Specifically, following Zhou et al.’s study (Xuefeng et al., 2021), the sample was divided into two groups based on the median of the centrality and structural holes representing the interlocking director network position, with the higher network position being the treatment group and the lower network position being the control group, and matched according to the 1:1 nearest neighbor matching method, with the matching variables containing all the control variables in Model 1. The matched sample was tested again and found that, after controlling for endogeneity caused by sample selectivity bias, interlocking director network position was still significantly and positively correlated with corporate risk-taking, again providing a robustness check for the previous findings (results omitted due to space constraints).

#### 4.5.2. The replacement of dependent variable

To further verify the robustness of this paper, the level of corporate risk-taking was re-measured (RISK3 and RISK4) with an observation period of 5 years (*t*–4, *t*) by referring to the study of He et al. (Ying et al., 2019). At the same time, referring to Su’s study (Kun, 2015), the volatility of stock returns (the logarithm of the standard deviation of annualized daily returns and the logarithm of the standard deviation of annualized weekly returns) was also used to re-measure corporate risk-taking (RISK5 and RISK6). Tables 6 and 7 show the regression results for the alternative risk-taking indicators. As can be seen from columns (1) to (6), the regression coefficients of the NET on the above risk-taking indicators are still significantly positive.

**Table 6:** Indicator sensitivity test: changing the observation period.

Name	Code	(1)	(2)	(3)	(4)	(5)	(6)
		RISK3	RISK4	RISK3	RISK4	RISK3	RISK4
Degree centrality	Degree	1.326^**^	3.459^***^				
		(2.42)	(2.60)				
Betweenness centrality	Betweenness			0.477^***^	1.206^***^		
				(2.62)	(2.70)		
Structural hole richness	SH					0.004^**^	0.011^***^
						(2.51)	(2.73)
Constant	Cons	0.364^***^	0.861^***^	0.366^***^	0.866^***^	0.363^***^	0.858^***^
		(9.39)	(9.26)	(9.45)	(9.32)	(9.37)	(9.24)
Control variables	Controls	Yes	Yes	Yes	Yes	Yes	Yes
Company FE	Company	Yes	Yes	Yes	Yes	Yes	Yes
Year FE	Year	Yes	Yes	Yes	Yes	Yes	Yes
Observations	N	16,315	16,315	16,315	16,315	16,315	16,315
Goodness of fit	Adj. R^2^	0.232	0.222	0.232	0.222	0.232	0.222
*F*-value	F	31.37^***^	32.52^***^	31.24^***^	32.37^***^	31.31^***^	32.52^***^

*p<0.1, **p<0.05, ***p<0.01.

**Table 7:** Indicator sensitivity test: volatility of stock returns.

Name	Code	(1)	(2)	(3)	(4)	(5)	(6)
		RISK5	RISK6	RISK5	RISK6	RISK5	RISK6
Degree centrality	Degree	1.548^**^	3.140^***^				
		(2.57)	(2.90)				
Betweenness centrality	Betweenness			0.953^***^	1.562^***^		
				(2.82)	(2.76)		
Structural hole richness	SH					0.002^**^	0.010^***^
						(2.45)	(2.90)
Constant	Cons	−2.773^***^	−1.548^***^	−2.781^***^	−1.556^***^	−2.775^***^	−1.551^***^
		(−23.59)	(−10.50)	(−23.65)	(−10.53)	(−23.63)	(−10.53)
Control variables	Controls	Yes	Yes	Yes	Yes	Yes	Yes
Company FE	Company	Yes	Yes	Yes	Yes	Yes	Yes
Year FE	Year	Yes	Yes	Yes	Yes	Yes	Yes
Observations	N	16,293	16,293	16,293	16,293	16,293	16,293
Goodness of fit	Adj. R^2^	0.580	0.490	0.580	0.490	0.580	0.490
*F*-value	F	1128^***^	780.9^***^	1134^***^	781.9^***^	1125^***^	780.2^***^

*p<0.1, **p<0.05, ***p<0.01.

#### 4.5.3. The replacement of regression model

The data used in this paper is an unbalanced panel data, which may face the problem of residual autocorrelation due to time trends in addition to the cross-sectional correlation problem at the company level. Therefore, in order to mitigate the influence of intra- and inter-group serial correlation problems on the regression results, this paper further adopted a more robust estimation method with two-way clustering of company and year for the *t*-values in the regression analysis. The details are shown in Table 8. The estimation results in columns (1)–(6) show that the empirical results remain consistent with the main regression results after using the two-way clustering of company and year.

**Table 8:** Two-way cluster analysis of company and year.

Name	Code	(1)	(2)	(3)	(4)	(5)	(6)
		RISK1	RISK2	RISK1	RISK2	RISK1	RISK2
Degree centrality	Degree	1.432^**^	2.713^**^				
		(2.12)	(2.14)				
Betweenness centrality	Betweenness			0.497^**^	0.929^**^		
				(2.33)	(2.34)		
Structural hole richness	SH					0.004^**^	0.008^**^
						(1.98)	(2.02)
Constant	Cons	0.459^***^	0.862^***^	0.460^***^	0.865^***^	0.458^***^	0.860^***^
		(4.48)	(4.45)	(4.48)	(4.45)	(4.47)	(4.45)
Control variables	Controls	Yes	Yes	Yes	Yes	Yes	Yes
Company FE	Company	Yes	Yes	Yes	Yes	Yes	Yes
Year FE	Year	Yes	Yes	Yes	Yes	Yes	Yes
Observations	N	19,689	19,689	19,689	19,689	19,689	19,689
Goodness of fit	Adj. R^2^	0.446	0.445	0.446	0.445	0.446	0.445

*p<0.1, **p<0.05, ***p<0.01.

## 5. Influence mechanism analysis

The above findings suggest that the interlocking director network can enhance corporate risk-taking, and the findings remain robust after a series of robustness tests. In order to further analyze the influence mechanism of NET on RISK, this paper uses stepwise regression and Sobel test to verify whether financing constraints (SA) and R&D investment (R&D) play a mediating role in the process of NET impact on RISK.

### 5.1. The mediating effect of SA

Models (7)–(9) are used in this paper to verify the mediating role of SA and R&D in the process of interlocking director network affecting corporate risk-taking. Table 9 reports the test results of the mediating effect of SA. Columns (1), (4), (7) show the regression results of the effect of interlocking director network on financing constraints, the regression coefficients of Degree, Betweenness and SH are significantly negative at the 1% level, indicating that the embedding of interlocking director network is beneficial to reduce the level of financing constraints. Columns (2), (5), (8) and (3), (6), (9) demonstrate the effects of Degree, Betweenness and SH on RISK1 and RISK2 after the inclusion of financing constraints, respectively. The coefficient of SA is significantly negative and the regression coefficients of Degree, Betweenness and SH are significantly positive and significantly smaller than the coefficients of the baseline regression results in Table 3, which indicates that there is a partial mediating effect of financing constraint between NET and RISK. In addition, in order to enhance the robustness of the results, the Sobel test is conducted on the basis of stepwise regression, and the test results are shown in the last row of Table 9. The *Z* value of the Sobel test is significantly positive (*p* < 0.01), which indicates that the mediating effect is robust. In summary, the interlocking director network can enhance corporate risk-taking by reducing financing constraints. Therefore, H2 is supported.

**Table 9:** Results of the mediating effect of SA.

Name	Code	(1)	(2)	(3)	(4)	(5)	(6)	(7)	(8)	(9)
		SA	RISK1	RISK2	SA	RISK1	RISK2	SA	RISK1	RISK2
Degree centrality	Degree	−6.261^***^	1.199^**^	2.276^**^						
		(−5.50)	(2.29)	(2.31)						
Betweenness centrality	Betweenness				−2.712^***^	0.396^**^	0.740^**^			
					(−6.13)	(2.25)	(2.25)			
Structural hole richness	SH							−0.026^***^	0.003^*^	0.006^*^
								(−8.07)	(1.90)	(1.91)
Financing constraints	SA		−0.037^***^	−0.070^***^		−0.037^***^	−0.070^***^		−0.037^***^	−0.070^***^
			(−3.40)	(−3.41)		(−3.38)	(−3.39)		(−3.39)	(−3.40)
Constant	Cons	3.609^***^	0.593^***^	1.114^***^	3.599^***^	0.594^***^	1.115^***^	3.613^***^	0.560^***^	1.112^***^
		(37.83)	(9.51)	(9.54)	(37.84)	(9.53)	(9.55)	(38.05)	(9.94)	(9.51)
Control variables	Controls	Yes	Yes	Yes	Yes	Yes	Yes	Yes	Yes	Yes
Company FE	Company	Yes	Yes	Yes	Yes	Yes	Yes	Yes	Yes	Yes
Year FE	Year	Yes	Yes	Yes	Yes	Yes	Yes	Yes	Yes	Yes
Observations	N	19,689	19,689	19,689	19,689	19,689	19,689	19,689	19,689	19,689
Goodness of fit	Adj. R^2^	0.872	0.251	0.248	0.873	0.251	0.248	0.873	0.251	0.248
F-value	F	953.9^***^	37.26^***^	37.79^***^	959.4^***^	37.20^***^	37.73^***^	966.2^***^	37.19^***^	37.72^***^
Sobel-test	Sobel Z		5.892^***^	5.915^***^		6.226^***^	6.256^***^		6.282^***^	6.310^***^
	Sobel Z-p		(0.000)	(0.000)		(0.000)	(0.000)		(0.000)	(0.000)

*p<0.1, **p<0.05, ***p<0.01.

### 5.2. The mediating effect of R&D

Table 10 shows the results of the mediating effect of R&D. The regression coefficients in columns (1), (4), and (7) are significantly positive, indicating that the interlocking director network can promote corporate R&D investment. Columns (2), (5), (8) and (3), (6), (9) demonstrate the regression results of the effect of NET on RISK when the mediating variable of R&D is added. The results show that the coefficients of R&D are significantly positive and the coefficients of the effects of Degree, Betweenness and SH on corporate risk-taking (RISK1 and RISK2) are reduced but are significantly positive at the 5% level (*p* < 0.05). In addition, the *Z* value of the Sobel test is significant at the 1% level (*p* < 0.01), which also demonstrates a partial mediating effect of R&D. Thus, interlocking director network can increase corporate risk-taking by increasing R&D investment. On this basis, H3 is supported.

**Table 10:** Results of the mediating effect of R&D.

Name	Code	(1)	(2)	(3)	(4)	(5)	(6)	(7)	(8)	(9)
		R&D	RISK1	RISK2	R&D	RISK1	RISK2	R&D	RISK1	RISK2
Degree centrality	Degree	5.809^***^	1.248^**^	2.361^**^						
		(9.96)	(2.38)	(2.39)						
Betweenness centrality	Betweenness				2.165^***^	0.428^**^	0.797^**^			
					(6.54)	(2.43)	(2.42)			
Structural hole richness	SH							0.017^***^	0.004^**^	0.007^**^
								(12.13)	(2.15)	(2.16)
R&D investment	R&D		0.032^**^	0.061^**^		0.032^**^	0.061^**^		0.032^**^	0.061^**^
			(1.99)	(2.03)		(1.97)	(2.02)		(2.01)	(2.06)
Constant	Cons	0.086^***^	0.456^***^	0.857^***^	0.093^***^	0.457^***^	0.859^***^	0.082^**^	0.455^***^	0.855^***^
		(2.69)	(11.47)	(11.45)	(2.92)	(11.49)	(11.48)	(2.54)	(11.46)	(11.44)
Control variables	Controls	Yes	Yes	Yes	Yes	Yes	Yes	Yes	Yes	Yes
Company FE	Company	Yes	Yes	Yes	Yes	Yes	Yes	Yes	Yes	Yes
Year FE	Year	Yes	Yes	Yes	Yes	Yes	Yes	Yes	Yes	Yes
Observations	*N*	19,689	19,689	19,689	19,689	19,689	19,689	19,689	19,689	19,689
Goodness of fit	Adj. *R*^2^	0.0561	0.250	0.247	0.0571	0.250	0.247	0.0545	0.250	0.247
F-value	*F*	25.68^***^	37.23^***^	37.82^***^	24.77^***^	37.19^***^	37.79^***^	29.93^***^	37.17^***^	37.77^***^
Sobel-test	Sobel Z		4.363^***^	4.470^***^		4.382^***^	4.495^***^		4.353^***^	4.457^***^
	Sobel Z-p		(0.000)	(0.000)		(0.000)	(0.000)		(0.000)	(0.000)

*p<0.1, **p<0.05, ***p<0.01.

## 6. Further analysis

To further explore the differences in the relationship between NET and RISK in different situations, this paper explores whether there are significant differences in the effects of NET on RISK under the influence of the nature of the ownership and the intensity of industry competition from the enterprise and industry levels.

### 6.1. Grouping test for the nature of ownership

The nature of ownership is an important factor influencing corporate risk-taking (Sila et al., 2016). Because of the difference in ownership, there is a significant difference in the ability to access resources between state-owned and private enterprises. Compared to state-owned enterprises (SOEs), non-SOEs are smaller, subject to a higher degree of financing constraints, have a greater need for resources, and therefore have a greater need to access resources by resorting to the informal system of interlocking director network. SOEs, although they are more likely to obtain resources for risk-taking behavior through the interlocking director network, also tend to adopt a more prudent investment strategy due to their more severe government intervention. Therefore, this paper argues that the nature of ownership will weaken the positive effect of NET on RISK.

To test whether the above analysis is true, following the method of Li et al. (Wenggui and Minggui, 2012), this study groups the nature of ownership (SOE) of the sample enterprises according to the nature of the ultimate controller. If the final controller of the enterprise is a state-owned entity, it is a state-owned enterprise (SOE = 1), or else SOE = 0. The grouping regressions are shown in Table 9. Columns (1)–(3) are the regression results for the state-owned enterprises group, and columns (4)–(6) are the regression results for the non-SOEs group. From the regression results in Table 11, it is clear that in the SOEs group, the regression coefficients of the indicators related to the interlocking director network are not significant, while in the non-SOEs group, the coefficients of Degree, Betweenness, and SH are significantly positive, which indicates that the nature of ownership weakens the positive effect of NET on RISK. Compared with state-owned enterprises, the effect of NET on RISK is more significant in non-SOEs.

**Table 11:** Further analysis of the nature of ownership.

Name	Code	RISK1					
		SOE = 1			SOE = 0		
		(1)	(2)	(3)	(4)	(5)	(6)
Degree centrality	Degree	0.326			1.743^**^		
		(0.55)			(2.15)		
Betweenness centrality	Betweenness		0.211			0.599^**^	
			(1.20)			(2.03)	
Structural hole richness	SH			0.002			0.005^**^
				(0.99)			(2.20)
Constant	Cons	0.403^***^	0.404^***^	0.403^***^	0.547^***^	0.548^***^	0.546^***^
		(7.50)	(7.51)	(7.51)	(9.42)	(9.43)	(9.41)
Control variables	Controls	Yes	Yes	Yes	Yes	Yes	Yes
Company FE	Company	Yes	Yes	Yes	Yes	Yes	Yes
Year FE	Year	Yes	Yes	Yes	Yes	Yes	Yes
Observations	*N*	7,588	7,588	7,588	12,101	12,101	12,101
Goodness of fit	Adj. *R*^2^	0.091	0.091	0.091	0.324	0.324	0.324
*F*-value	*F*	7.482^***^	7.392^***^	7.492^***^	39.58^***^	39.30^***^	39.29^***^

*p<0.1, **p<0.05, ***p<0.01.

### 6.2. Grouping test for the intensity of industry competition

Corporate risk-taking is also influenced by the intensity of industry competition. On the one side, the industry competition can reduce the internal and external information asymmetry of enterprises, help enterprises to effectively unblock and improve the collection and transmission channels of relevant information, and alleviate the agency conflict of enterprises; on the other side, the fierce industry competition will accelerate the rapid flow of information between industries, reduce the level of financing constraints and increase the level of risk-taking of enterprises. The more competitive the industry is, the more complex the environment in which the enterprise is located. The interlocking director network can help companies cope with the pressure brought by industry competition. Therefore, this paper argues that the intensity of industry competition will promote the positive effect of NET on RISK.

To test whether the above analysis is correct, this study refers to the academic operation of Du and Ma (Shanzhong and Lianfu, 2022), using the Herfindahl–Hirschman Index (HHI) to measure the intensity of industry competition. The larger the HHI, the lower the industry competition. Specifically, the HHI is calculated by the sum of squares of the proportion of the operating income of listed companies in the industry in the total operating income of the industry. Then, using the annual median as the boundary, samples larger than the annual median are classified as the group with low industry competition (HHI = 1), and samples smaller than the median are defined as the group with high industry competition (HHI = 0), and the results of the grouping test are shown in Table 12. Columns (1)–(3) are the regression results for the group with low industry competition, and columns (4)–(6) are the regression results for the group with high industry competition. The regression results in Table 12 show that the coefficients of Degree, Betweenness, and SH are significantly positive in the group with high industry competition, while the coefficients of the indicators related to interlocking director network are not significant in the group with low industry competition, indicating that the enhancement of NET on RISK is more pronounced when industry competition is high.

**Table 12:** Further analysis of the intensity of industry competition.

Name	Code	RISK1					
		HHI = 1			HHI = 0		
		(1)	(2)	(3)	(4)	(5)	(6)
Degree centrality	Degree	0.344			2.169^***^		
		(0.48)			(2.98)		
Betweenness centrality	Betweenness		0.124			0.797^***^	
			(0.51)			(3.14)	
Structural hole richness	SH			0.002			0.005^**^
				(0.97)			(2.14)
Constant	Constant	0.494^***^	0.494^***^	0.494^***^	0.488^***^	0.491^***^	0.486^***^
		(8.78)	(8.80)	(8.80)	(7.57)	(7.60)	(7.52)
Control variables	Controls	Yes	Yes	Yes	Yes	Yes	Yes
Company FE	Company	Yes	Yes	Yes	Yes	Yes	Yes
Year FE	Year	Yes	Yes	Yes	Yes	Yes	Yes
Observations	*N*	11,210	11,210	11,210	8,479	8,479	8,479
Goodness of fit	Adj. *R*^2^	0.255	0.255	0.255	0.242	0.242	0.241
*F*-value	*F*	22.17^***^	22.16^***^	22.15^***^	16.51^***^	16.44^***^	16.49^***^

*p<0.1, **p<0.05, ***p<0.01.

## 7. Conclusion and insights

Based on the social network embeddedness perspective, this paper selects Chinese A-listed companies in Shanghai and Shenzhen from 2007 to 2020 as the research sample to empirically examine the influence mechanism of NET on RISK. Consistent with previous studies (Larcker et al., 2013; Min et al., 2015), we find that NET does significantly improve the level of RISK, and this finding still holds after robustness tests using the instrumental variables method, changing the regression model and replacing the dependent variable. In terms of the influence mechanism, the NET enhance RISK by reducing SA and increasing R&D. Further discussion reveals that the effect of NET on RISK is more pronounced in non-SOEs and enterprises with high industry competition than in SOEs and enterprises with low industry competition.

The research in this paper provides the following insights into the construction of inter-enterprise interlocking director network and the enhancement of risk-taking level: (1) Listed companies should pay attention to the construction of interlocking director network and make full use of their unique “resource effect” and “governance effect” to help companies obtain external resources and network information required for risk-taking and further enhance corporate risk-taking level. (2) The government and relevant departments should encourage and guide enterprises to build a reasonable interlocking director network system and further optimize its resource allocation function to alleviate the financing constraints faced by enterprises and promote their R&D investment intensity. (3) Due to the fact that the function of interlocking director network is more significant in non-SOEs and enterprises with high industry competition. Therefore, enterprises should also reasonably select and use the interlocking director network according to their own situation and the external market competition environment they are in.

Still, our study has certain limitations on which further research will be explored. First, we failed to comprehensively consider the differences in the role of different types of interlocking director networks. Interlocking directors can be divided into independent directors and non-independent directors, and the impact of the two on corporate risk-taking may differ. Future research can further subdivide the interlocking director network into independent director network and non-independent director network for comparative research. Second, this study lacks further exploration of the intrinsic relationship between the two. The interlocking director network may not only improve corporate risk-taking through the two channels of reducing financing constraints and increasing R&D investment, but there may also be other paths of action between the two. We will further explore the influence mechanism between the two in the future. Third, we did not verify the differential effects of other types of social networks on corporate risk-taking. There are various ways of forming social network relationships among firms, but in this paper, we only considered interlocking director network relationships embedded among firms. Future research can examine the impact of multiple types of social networks on corporate risk-taking. Fourth, the research conclusions of this paper are based on the “static” basis, without considering the impact and mechanism of NET on RISK under the dynamic situation. In future research, we can establish a dynamic model to break the bottleneck of horizontal comparison and make the research results more consistent with the actual situation.

## Data availability statement

The original contributions presented in the study are included in the article/supplementary material, further inquiries can be directed to the corresponding author.

## Author contributions

HL and YL: conceptualization and data collection, and writing—original draft preparation. HL: formal analysis. YL: software and validation. HL and QS: funding acquisition, and writing—review and editing. All authors contributed to the article and approved the submitted version.

## Funding

This research was funded by the Natural Science Foundation of China (No. 71771112), and Project of Liaoning Provincial Federation Social Science Circles of China (No. L20BGL047).

## Conflict of interest

The authors declare that the research was conducted in the absence of any commercial or financial relationships that could be construed as a potential conflict of interest.

## Publisher’s note

All claims expressed in this article are solely those of the authors and do not necessarily represent those of their affiliated organizations, or those of the publisher, the editors and the reviewers. Any product that may be evaluated in this article, or claim that may be made by its manufacturer, is not guaranteed or endorsed by the publisher.
